# Advances of RRAM Devices: Resistive Switching Mechanisms, Materials and Bionic Synaptic Application

**DOI:** 10.3390/nano10081437

**Published:** 2020-07-23

**Authors:** Zongjie Shen, Chun Zhao, Yanfei Qi, Wangying Xu, Yina Liu, Ivona Z. Mitrovic, Li Yang, Cezhou Zhao

**Affiliations:** 1 Department of Electrical and Electronic Engineering, Xi’an Jiaotong-Liverpool University, Suzhou 215123, China; Zongjie.Shen@xjtlu.edu.cn (Z.S.); Yanfei.Qi01@xjtlu.edu.cn (Y.Q.); Cezhou.Zhao@xjtlu.edu.cn (C.Z.); 2 Department of Electrical Engineering and Electronics, University of Liverpool, Liverpool L69 3BX, UK; Ivona@liverpool.ac.uk; 3 School of Electronic and Information Engineering, Xi’an Jiaotong University, Xi’an 710061, China; 4 College of Materials Science and Engineering, Shenzhen University, Shenzhen 518060, China; wyxu@szu.edu.cn; 5 Department of Mathematical Sciences, Xi’an Jiaotong-Liverpool University, Suzhou 215123, China; Yina.Liu@xjtlu.edu.cn; 6 Department of Chemistry, Xi’an Jiaotong-Liverpool University, Suzhou 215123, China; Li.Yang@xjtlu.edu.cn

**Keywords:** artificial intelligence, thin film, 2D materials, switching mechanisms, bionic synaptic application, RRAM

## Abstract

Resistive random access memory (RRAM) devices are receiving increasing extensive attention due to their enhanced properties such as fast operation speed, simple device structure, low power consumption, good scalability potential and so on, and are currently considered to be one of the next-generation alternatives to traditional memory. In this review, an overview of RRAM devices is demonstrated in terms of thin film materials investigation on electrode and function layer, switching mechanisms and artificial intelligence applications. Compared with the well-developed application of inorganic thin film materials (oxides, solid electrolyte and two-dimensional (2D) materials) in RRAM devices, organic thin film materials (biological and polymer materials) application is considered to be the candidate with significant potential. The performance of RRAM devices is closely related to the investigation of switching mechanisms in this review, including thermal-chemical mechanism (TCM), valance change mechanism (VCM) and electrochemical metallization (ECM). Finally, the bionic synaptic application of RRAM devices is under intensive consideration, its main characteristics such as potentiation/depression response, short-/long-term plasticity (STP/LTP), transition from short-term memory to long-term memory (STM to LTM) and spike-time-dependent plasticity (STDP) reveal the great potential of RRAM devices in the field of neuromorphic application.

## 1. Introduction

**Figure 1:**
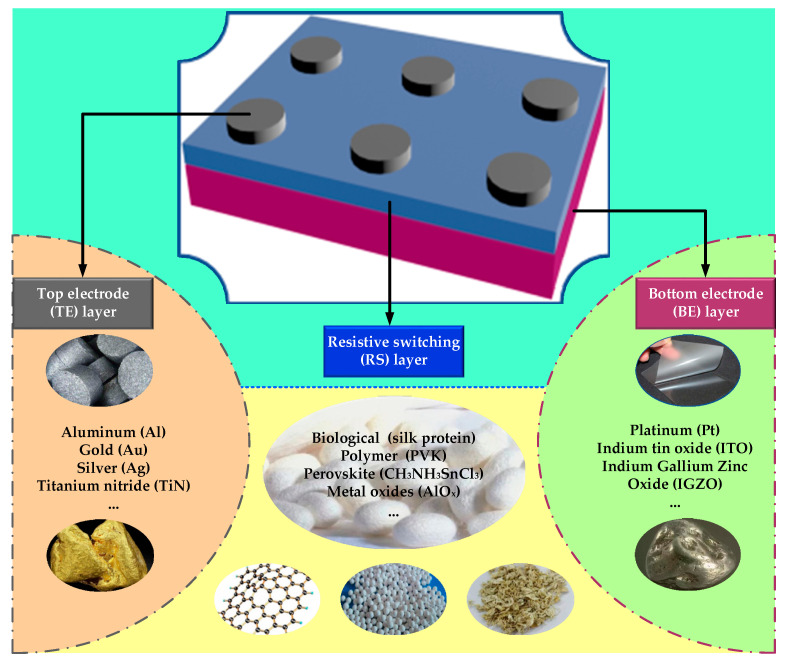
Illustration of sandwich structure for RRAM devices.

In the neuromorphic system of the human brain, neuromorphic synapses are believed to be responsible for transmitting biological information. As the most typical and exquisite representative of the biological memory system, the human brain can store and process massively biological information with the adjustment of synaptic connection strength (synaptic weight) [1]. Meanwhile, they also provide complicated orthosympathetic space and energy balance [1,2]. In general, a biological synapse with the structure of dendrite, axon terminal and synaptic cleft is identified as a neuron linker that can permit a neuron to transmit neurotransmitters to another adjacent neuron [2]. For now, artificial synapse has attracted extensive interests and attention as the research focus in the development of artificial intelligence (AI) industry, which mainly concentrates on biomimetic synaptic functions simulated by memory functional devices in the computer industry [2]. As one of the most critical carriers for the inheritance of human civilization and the development of information technology, volatile and non-volatile memory (NVM) devices have always acted as dominating components of the development of memory devices. In the trend of miniaturization of electronic equipment, the demand for memory devices with small size, low voltage, low power consumption, and superior performance has been under extensive consideration. Currently, silicon-based flash memory, which dominates the market of data storage devices, has been difficult in meeting the needs of future development of data storage devices due to its physical and technological limitations, such as high operation voltage, high power consumption and low retention capacity [3,4,5,6]. As one of the emerging technologies of NVM, resistive random access memory (RRAM) device has been given attention as one of the next-generation memory devices. Simmons et al. reported a resistive switching (RS) characteristic in the memory device with the structure of Au/SiO_2_/Al as early as in 1967, which provided the theoretical and experimental foundation of RRAM [7]. They demonstrated the new type of memory with the simple sandwiched structure, including top electrode (TE) layer, bottom electrode (BE) layer and an intermediate functional thin film layer (RS layer), which can be observed in Figure 1. This simple structure consisting of conductor/semiconductor or insulator/conductor makes RRAM cells be integrated into the passive crossbar array easily, with the size as small as 4F^2^ (F—the minimum feature size), which can be evenly divided into n parts (4F^2^/n) in the vertically stacked three-dimensional (3-D) architectures (n means the stacking layer number of the crossbar array) [1,2,3].

The simple sandwich structure of RRAM device indicates the significance on thin film materials investigation and application. In general, performance of a RRAM device largely depends on characteristics of thin film materials for electrode and RS layers. As the main conducting medium, top and bottom electrodes are considered to enhance the electrical conductivity of RRAM devices [2]. Therefore, some thin film materials with good electrical conductivity are then chosen as candidates of the electrode, such as pure metal, semiconductor and graphene materials [1,2,3]. For most bipolar RRAM devices, reactive metal like titanium (Ti) [8], nickel (Ni) [9], copper (Cu) [10] and silver (Ag) [11] are always used as TE due to their high metal activity. Compared with reactive metal, where Platinum (Pt) [12] always acts as BE in order to provide activity variation of RRAM devices. In addition, semiconductor materials like heavily doped silicon and indium tin oxide (ITO) are always chosen as electrodes due to their high electrical conductivity [13,14]. Currently, 2D thin film materials such as graphene and graphene oxide (GO) are also attracting increasing attention as candidates of electrode because of the excellent mobility of carriers and high thermal/electrical conductivity [15,16,17]. Compared with electrode thin film materials, more researchers focus on thin film materials applicated on RS medium, and inorganic materials are the main investigation objectives, including oxides and solid electrolyte, which will be discussed in Chapter 3 [11,18,19,20,21,22,23,24,25,26,27]. 

The performances of thin-film-material-based RS layers have a decisive influence on the performance of RRAM devices, which indicates that the fabrication methods of RS layers or synthesis technologies of thin film materials cannot be neglected [1,2,3,4,5,6]. Currently, several main fabrication technologies for RS layers have received extensive recognition by researchers, such as atomic layer deposition (ALD), magnetron sputtering, chemical vapor deposition (CVD) and solution-processed deposition. For most inorganic thin film materials used on RRAM devices (such as metal oxides and solid electrolyte), ALD and sputtering are two of the most advanced technologies due to stable performance of RS layers fabricated accordingly [5,6,7,8,9,10,11]. In addition, our previous study indicated that some other fabrication techniques for 2D thin film materials (such as graphene, GO and hexagonal boron nitride (h-BN), h-BN is considered as one of the most promising 2D materials with the function as 2D insulating template for high performance 2D electronic and photonic devices) have also been under intensive consideration, including nucleation and growth, liquid phase exfoliation and electrochemical exfoliation [21]. 

**Figure 2:**
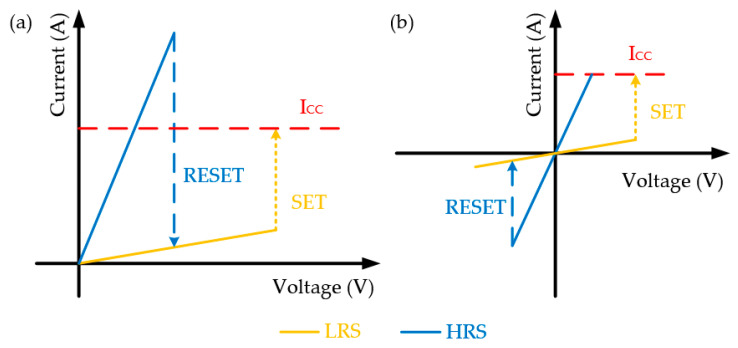
(**a**) Unipolar and (**b**) bipolar Modes for RRAM devices.

Apart from its typical physical structure, two basic switching states related to the conductive filament (CF) of RRAM devices, OFF and ON states, are commonly electrically characterized, which are also referred to as high-resistance-state (HRS) and low-resistance-state (LRS), respectively. With the transformation of resistance states, RRAM devices can complete the data storage process based on ‘0’ or ‘1’. HRS value of the device shows the low conductance state while the device demonstrates a high conductance state with LRS value. ON/OFF ratio is determined by the ratio between HRS and LRS. With the applied voltage bias, the SET operation is defined from HRS to LRS and the RESET operation is the transition from LRS to HRS. Stop voltages of SET and RESET process are defined as V_SET_ and V_RESET_. In general, two different switching types are defined as unipolar and bipolar [28,29,30], as illustrated in Figure 2. The unipolar switching mode is defined by the amplitude of the applied voltage bias while bipolar switching depends on the polarity of the applied voltage bias. In addition, as one of NVM devices, the endurance and retention properties of RRAM are also the presence of device reliability [4,5,6,7,28,29,30]. 

Compared with conventional silicon-based memory devices like flash memory, it is noted that RRAM devices have demonstrated a series of advantages such as low operation voltage, low power consumption, high density, and enhanced compatibility with traditional complementary metal oxide semiconductor (CMOS) technology [31,32,33]. In addition, with the deepening of research on artificial intelligence (AI) hardware equipment, biomimetic synapse behaviors of RRAM devices have also received extensive attention, which has non-negligible influence in the investigation of electrical artificial synapse [15,17,34,35,36,37,38]. However, some other limitations and challenges of RRAM devices cannot be neglected, such as synthesis methods of RS materials, stability of device performance and storage mechanism of devices with different materials. 

In this work, we provide a review of research for several main aspects, including exploration on switching mechanisms, investigation of thin film materials of RRAM devices and bionic synaptic application of RRAM devices. Section 1 provides an overview of background induction for RRAM devices. Section 2 will demonstrate a detailed discussion of different switching mechanisms. Section 3 will focus on various thin film materials applied to RRAM devices including RS medium materials and electrode materials. Section 4 will show an investigation on the neuromorphic application for RRAM devices, and Section 5 will be the conclusion of this review.

## 2. Resistive Switching (RS) Mechanisms of RRAM Devices

To present a comprehensive overview of RRAM devices, firstly, it is necessary to implement in-depth survey on different RS mechanisms of RRAM devices, which is still a controversial issue. The current investigation of RS mechanisms for sandwich structure RRAM devices is related to not only materials selection of electrode/RS medium but also utilized operation modes. For now, the most widely recognized switching mechanism is based on conductive filaments (CFs). However, there is no uniform and standard answer for some important issues about CFs with microscope chemical composition, physical morphology and the formation/rupture process, which have an intensive relationship to performance and working principle of RRAM devices. In this work, we will focus on the research of several working mechanisms related to anion/cation migration and thermal-chemical reaction, including thermal-chemical mechanism (TCM), valance change mechanism (VCM) and electrochemical metallization (ECM). 

### 2.1. Thermal-Chemical Mechanism (TCM)

**Figure 3:**
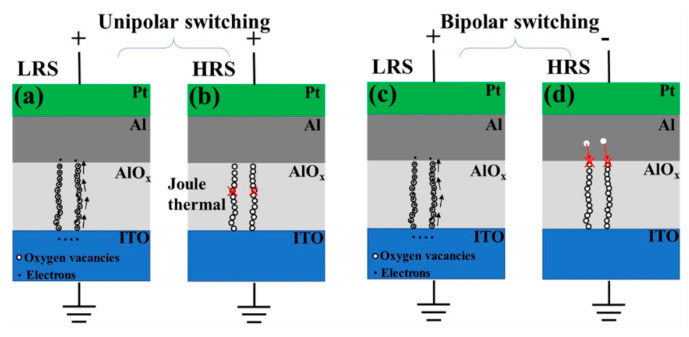
Switching mechanism of unipolar (**a**,**b**) and bipolar (**c**,**d**) AlO_x_-based RRAM devices, reproduced from [39], with permission from Springer Nature, 2020.

Theory about TCM can be applied to explain the formation and fracture of CFs resulted from ions (oxygen ion or metal ion) migration induced by thermal-chemical reaction (Joule heating), which is independent of the switching mode (unipolar and bipolar) for RRAM devices [39,40,41]. Zhang et al. explained the working principle of their Pt/Al/AlO_x_/ITO RRAM device with TCM theory [39]. As illustrated in Figure 3, oxygen ions driven by Joule heating effect drifted to TE and left oxygen vacancies in the AlO_x_ layer; consequent CFs based on the accumulation of oxygen vacancies set the device to LRS. For the RESET process of unipolar device, the current steadily increased with the increasing positive voltage bias, the formed CFs finally broke when it reached the critical temperature induced by Joule heating, which made the device switch back to HRS. Similarly, for the RESET process of bipolar device, the oxygen ions drifted back to the AlO_x_ layer due to the melting of CFs and switched the device to HRS again. TCM based on Joule heating reaction is related to the formation and rupture of CFs. Tsuruoka et al. also proposed a research report on Ag/Ta_2_O_5_/Pt RRAM device with TCM based on the Joule heating effect [40]. The filament based on metal Ag played a dominating role during the RS process. The formation of Ag CF made the device from HRS to LRS during the SET process. Due to the Joule-heating-based oxidation, the Ag CF ruptured by the thermal dissolution and completed the RESET operation. In general, with the SET/Forming operation, the thermal decomposition process that occurrs in the RS medium generates the ions migration in the RRAM device and the resulting formation process of CFs transforms the device from HRS to LRS. With the reversed voltage bias applied onto the electrode, the existing CFs rupture due to the thermal melting reaction, which transforms the device back to HRS and completes the RESET process accordingly.

### 2.2. Valance Change Mechanism (VCM)

**Figure 4:**
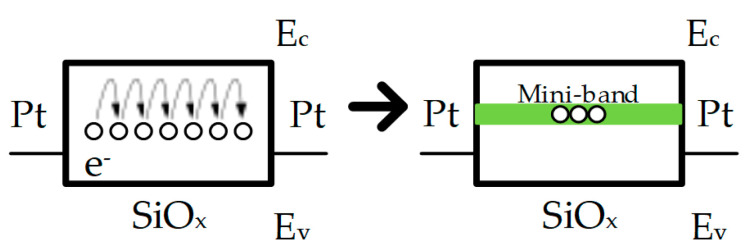
Schematic of discontinuous and continuous states of mini-band DBs in the middle of silicon oxide band gap.

Unlike the TCM mentioned above, VCM has oxygen-related defects/vacancies and their electrochemical reaction occurred in the RS medium [42,43,44,45,46,47]. In addition, it is not necessary for an RRAM device to operate with the structure that consists of an active electrode and an inert electrode, namely, the activity difference between TE and BE is not required [48]. Chen et al. researched the unipolar performance of Pt/SiO_x_/Pt RRAM device with VCM and dangling bond (DB) [43]. As illustrated in Figure 4, with the effect of external electric field, the strength of the polar covalent Si–O bond was weakened and finally broken. With the much higher concentration of DB near the middle of the silicon band gap, the hopping process could make the transportation of the electron through the discontinuation of DB, which was similar to the initial state (HRS) of their devices. If the DB concentration arose up to the threshold value of the percolation path, the electron transport could occur in the mini-band of DBs and the device switched into LRS, which accordingly indicated the SET process. Munjal also initiated the analysis process of CoFe_2_O_4_-based RRAM devices with the VCM theory [47].

In most cases, for VCM RRAM devices, the resistance change performance is attributed to the formation and rupture process of CF based on oxygen vacancies in the RS layer [42,43,44,45,46,47]. With the positive voltage bias applied onto the inert electrode, oxygen ions drift from where they stayed before with the effect of external electric field and oxygen vacancies left in the RS medium. The consequent CF path made up of leftover oxygen vacancies connects TE and BE through the functional layer, which increases the electric conductivity of the RS thin film and switches the device from HRS to LRS. Whereas, with the reversed voltage bias onto the same electrode, oxygen ions drift back to the RS layer and result in the rupture of formed CF, which makes the device switch back to HRS again. Therefore, oxygen defects/vacancies and oxygen may be the dominating aspect during the growth and destruction of CF in the functional layer. 

### 2.3. Electrochemical Metallization (ECM)

Compared with TCM and VCM, ECM based on electrochemical reaction and cation migration, as the most recognized mechanism, is always used to explain the working principle of RRAM device with an active electrode, which is similar to VCM [49,50,51,52,53,54,55]. Generally, most active electrodes for ECM devices are active metal such as Cu [50,51,52] and Ag [49,53,54,55]. Tsuruoka et al. investigated Cu/Ta_2_O_5_/Pt RRAM device based on Cu filaments [50]. With a positive voltage bias applied onto Cu TE, Cu atoms near the interface between Cu layer and Ta_2_O_5_ layer were dissolved into Cu ions (Cu^2+^) and electron (e^−^) due to the electrochemical reaction. These Cu^2+^ ions drifted towards the RS layer with the effect of external electric field, which induced the Cu^2+^ ions supersaturation near the Ta_2_O_5_/Pt interface. Then continuous cathodic deposition reaction occurred between Cu^2+^ and e^−^ led the formation of Cu-based filament and switched the device into ON state. 

**Figure 5:**
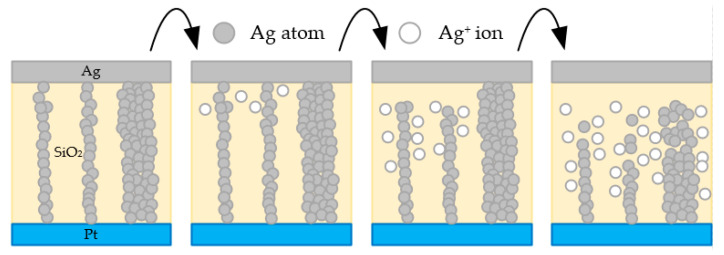
Illustration of multilevel RESET process of Ag/SiO_2_/Pt RRAM device.

Yu et al. also confirmed that multiple Ag filaments attributed to the multilevel RESET behavior of RRAM device with a switching layer based on nonmetal materials (Ag/SiO_2_/Pt) [49]. With a small negative voltage bias, the Ag/SiO_2_/Pt device exhibited gradual resistance increasement. When the voltage bias continued to increase beyond a threshold value, the resistance of device was increased to a higher state sharply, which suggested that multiple Ag filaments were effective as predicted. As demonstrated in Figure 5, in the SET process, Ag CFs with different sizes existed under a big CC after several switching cycles. During the RESET process, Ag from Ag CFs transferred into Ag^+^ due to the dissolution reaction, which resulted in a gradual resistance increase of device. When these smaller CFs were broken, the resistance changed significantly. After that, CFs with larger sizes were getting thinner until they ruptured, which further induced the multilevel performance of RESET process. Long et al. also used ECM based on Ag filament to explain the switching mechanism of Ag/ZrO_2_: Cu/Pt RRAM device [53]. With the effect of external electric field induced by voltage bias applied onto TE Ag, the oxidation process occurred on Ag atoms and Ag atoms transferred into Ag ions (Ag → Ag^+^ + e^−^). Then Ag^+^ migrated gradually to BE Pt as the electric field increased in the ZrO_2_ thin film and ions were reduced back to atoms (Ag^+^ + e^−^ → Ag). Finally, the formed Ag filament switched the device into LRS when the voltage reached V_SET_, which showed the related metallic transportation behavior. However, for the RESET process, when the voltage bias with reversed polarity was applied onto the active electrode, the existing Ag filament was broken within the oxide layer due to the electrochemical reaction w/o Joule heat assistance. Similarly, research reported by Tsuruoka et al. also presented the same perspective, which indicated that the RESET process related to formed metallic filaments might be related to electrochemical reaction w/o Joule heat assistance [50]. 

### 2.4. Modeling Analysis for Switching Mechanisms

In order to provide convincing explanations about switching mechanisms, some researchers focused on establishing analytical models during the investigation process [41,56,57,58,59,60,61]. Ren et al. provided a specific explanation on the RS mechanism of the Al/CH_3_NH_3_PbI_3_/FTO RRAM device, which was mainly based on a physics-based analytical model with mathematical equations [56]. In this model, the RS mechanism was generally attributed to the migration of iodine vacancy (V_I_) driven by electric field, which mainly includes operation phases such as electroforming, RESET and SET transitions. During the electroforming process, V_I_ nucleation occurred, induced by external electric field, and then CFs were formed in the perovskite film with the voltage bias applied onto the TE. They simplified the CF as a cylinder with radius (r) and height (h) and the obvious boundary existed between CF and outer region. In general, the region of the device where CF itself existed was the LRS region while other regions without CF indicated the HRS region. As illustrated in Figure 5, CFs themselves formed by Ag atoms (gray dots in the figure) represented LRS region while the pure wheat region without any gray dots indicated the HRS regions without CFs. With the combination of equations of Fick and Soret diffusion and related carrier drift theory, operations of RESET and SET were simulated during the switching process. 

During the RESET process, V_I_ migrated from TE to BE due to the existence of Fick diffusion, which could be regulated by equations:

(1) $$J_{\mathrm{Fick}}=D\nabla n$$

(2) $$D=0.5\alpha^{2}f\;exp\left(-E_{a}/kT\right)$$

where *D* was the diffusivity coefficient, ∇n was the concentration gradient of V_I_; α, f, $E_{a}$, *k* and *T* were vacancy hopping distance, escape-attempt frequency, migration activation energy, Boltzmann constant and local temperature, respectively. The gradient direction was always from BE to TE due to the function of V_I_ reservoir for BE, thus the RESET process was expected to be confined by the Fick diffusion. Apart from Fick diffusion, another vital factor, the voltage-field activated V_I_ drift could not be neglected, which could be expressed by equations:

(3) $$J_{drift}=-vn$$

(4) $$v=af\;exp\left(-E_{a}/kT\right)sinh\left(qaE/2kT\right)$$

where *v* was the drift velocity, *E* was the electric field inside the CF, and *q* was elementary charge. With the combination of *J_Fick_* and *J_drift_*, the time evolution of CF size could be expressed by equation:

(5) $$\frac{dg}{dt}=D\alpha_{1}e^{-\frac{\beta_{1}}{0.5\left(L-g\right)}}-v$$

where *L* is the thickness of perovskite film, and *g* is the gap length. In addition, the Joule heating flow that occurred in the lateral direction of CF was also noted, which had a closed relationship with the length and diameter of CF. For the SET process, V_I_ migration occurred from BE to TE with the effect of reversed applied voltage bias resulting in the refill of the gap region. They explained the phenomenon with a set kinetics model based on Fick and Soret diffusion. Similar to Fick diffusion in the RESET process, the formation and growth of CFs were also suppressed by Soret diffusion in SET process, which was associated with the migration tendency of V_I_ towards the region with higher temperature. 

According to their simulation results, the electric field inside CF decreased gradually when the depletion process occurred and then induced the slowing down of gap formation, which was used to explain why the growth of gap length mainly occurred in the positive part of voltage sweep. Apart from mathematical modeling, another main technique is modeling with software to justify the switching mechanisms. Software like COMSOL was also used to establish physics-based analytical models for further analysis [41,60]. Zhou et al. proposed a COMSOL-based model to research the switching mechanism of the Ti/HfO_2_/TiN RRAM device [60]. They chose the 1st, 10th and 20th cycles as fitting analysis objectives, which all performed with Schottky emission. Based on the Schottky fitting formula, the gradual larger Schottky distance and the barrier had a relationship with a larger intercept and the slope of Ln(I)-(V)^1/2^. According to the simulation results, the strongest electric field that existed in the tip of CF contacted the electrode in the initial SET process. After that, with more SET and RESET operations, oxygen ions accumulation occurred in the interface between TiN and HfO_2_ layers, which made SET and RESET more difficult to achieve. Sun et al. also reported a similar mechanism on Cu/ZrO_2_/Pt RRAM device [41].

## 3. Thin Film Materials of RRAM Devices

Apart from different RS switching mechanisms, most researches also put effort into the study of various thin film materials applied in RRAM devices. Before we present an investigation of materials application, it is necessary to provide brief induction of figures of merit (FoMs), which are mainly used to assess the performance of RRAM devices, including operation speed, power consumption, reliability, scalability, and cost [62]. Operation speed is defined by random-access time and effective time of write & erase (w & e) speed of a single RRAM cell; power consumption is affected by static and dynamic power consumption; reliability consists of endurance; retention properties are used to determine whether an RRAM device is reliable; scalability of an RRAM device determines whether RRAM devices can be developed in line with current trends of increasing device density; and the cost directly has an impact on marketization or mass-production of the proposed devices [62]. Apart from the main FoMs presented above, some other specific characteristics of FoMs such as SET/RESET voltage, ON/OFF switching ratio, distribution of operation voltage, and resistance cannot be neglected accordingly. 

Thin film materials application of RRAM devices can be divided into two directions: materials for RS medium and materials for electrode (TE/BE). Materials of RS medium play a decisive role in the switching process, which has a direct and significant influence on the performance of RRAM devices such as ON/OFF ratio and device stability. On the other hand, electrode materials of RRAM devices more affect switching modes of RRAM devices, which should also be under further investigation [13,14,18,48,63,64]. 

### 3.1. Thin Film Materials of RS Medium

**Figure 6:**
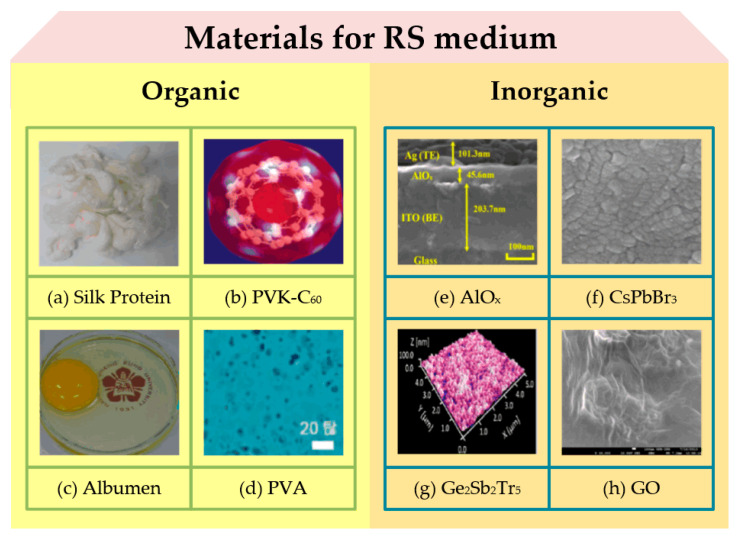
Various thin film materials of RS medium for RRAM devices. (**a**) Silk protein, reproduced from [65], with permission from John Wiley and Sons, 2016. (**b**) PVK-C_60_, reproduced from [72], with permission from American Chemical Society, 2007. (**c**) Albumen, reproduced from [67], with permission from Springer Nature, 2015. (**d**) PVA, reproduced from [71], with permission from John Wiley and Sons, 2011. (**e**) AlO_x_, reproduced from [19], with permission from Elsevier, 2020. (**f**) CsPbBr_3_, reproduced from [73], with permission from John Wiley and Sons, 2019. (**g**) Ge_2_Sb_2_Tr_5_, reproduced from [38], with permission from John Wiley and Sons, 2019. (**h**) GO, reproduced from [74], with permission from AIP Publishing, 2013.

A lot of thin film materials have been researched as RS mediums of RRAM devices due to their exhibition of RS characteristics with the effect of external electric field. In general, organic materials and inorganic materials are two main categories of RS medium, as illustrated in Figure 6. Research of RS medium based on organic materials mainly focuses on biological materials (silk protein/fibroin, nanocellulose and albumen) [65,66,67], polymer materials (PVK (polyvinyl carbazole), PVA (polyvinyl alcohol), PDA (Polydiacetylene), PTH (polythiophene)) [68,69,70,71], and other materials. Chen et al. presented an RRAM device fabricated with spin-coated chicken egg albumen layer [67]. With the low SET/RESET voltage ~3 V, the reliable switching endurance was observed over 500 cycles with ~10^3^ ON/OFF ratio. Similarly, Wang et al. reported an RRAM device with a structure of Au/Mg/fibroin/Mg and the fibroin-based RS layer was fabricated with drop-casting method [65]. Their device exhibited excellent performance with operation voltage lower than ~2 V, ON/OFF ratio higher than ~10^3^ and retention time longer than ~10^4^ s. In addition, they evaluated the transient behaviors of RRAM devices with the immersion process in DI water, which indicated the great potential of silk fibroin applied to transient and biocompatible electronics. Although the increasing number of research studies has demonstrated the feasibility of RRAM devices with organic materials, high power consumption under high operation voltage and dispersed voltage/resistance distribution induced by disappointing stability and reliability of device fabricated with organic materials indicated that it is necessary to further investigate perfect application of organic materials in the industry of memristive devices. In the future, most of these organic materials will be considered as probable candidates of application in flexible and wearable memristive devices with health diagnosis monitoring as the main function.

Compared with RRAM devices fabricated with organic materials, better electrical performance of RRAM devices based on inorganic materials can be observed with more stable switching behavior, lower energy consumption and longer retention time. Inorganic materials are also being given extensive attention due to their simple manufacturing process and superior properties. In this review, we will present RRAM-related inorganic materials with oxides, solid electrolyte and two-dimensional (2D) materials. Table 1 demonstrated a performance comparison among RRAM devices with different oxide layers, including binary and complex oxide materials. As one of the most typical representatives of oxides, binary oxides have been explored for over half a century due to their simple composition, low manufacture cost, compatibility with traditional CMOS (complementary metal-oxide-semiconductor transistor) technology, and ease of fabrication and control. Among binary oxides, binary metal oxides such as Al_2_O_3_ [9,20,75], NiO [8,12,76], TiO_2_ [21,77,78], HfO_2_ [60,79,80], ZnO [81,82,83], and ZrO_2_ [22,84,85] are always playing main roles in materials application of RS medium.

The first report of RS performance in binary metal oxides was proposed by Hickmott in 1962 [91], which demonstrated the RS characteristics of Al/Al_2_O_3_/Al device under the effect of an electric field. With the development of fabrication methods for electronic devices with thin films, binary metal oxides thin films fabricated by sputtering, ALD (atomic layer deposition) and solution-processed methods have received more interest due to their superior performance. Also, it is indicated that some researchers not only focus on RS layer fabricated with conventional pure binary metal oxides, but also explore effective ways of optimization treatment on metal-oxides-based RS layers, such as stack layers, ionic liquid (IL) process and nanoparticle (NP) doping process, and other similar treatment methods [8,20,32].

**Figure 7:**
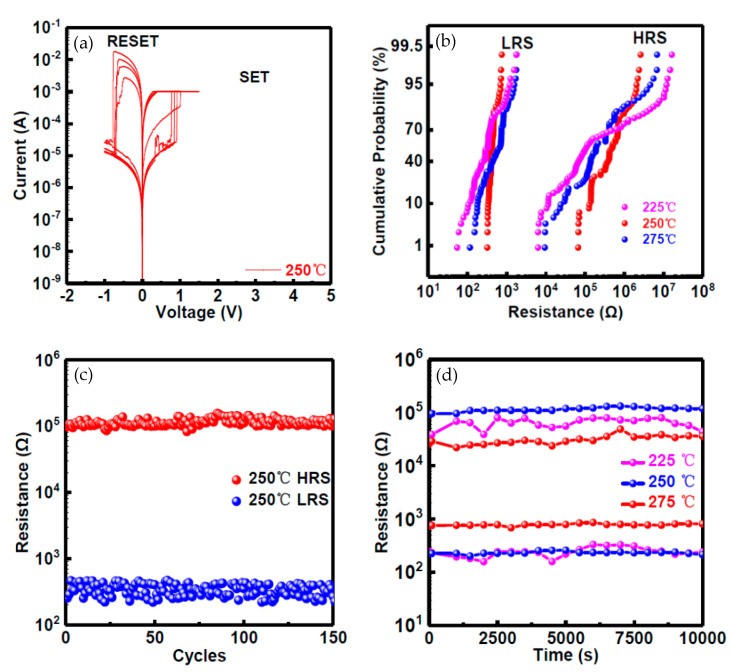
RS performance, including (**a**) bipolar I-V characteristic, (**b**) resistance distribution, (**c**) endurance and (**d**) retention performance, of Ni/solution-processed AlO_x_/Pt RRAM devices annealed at different temperatures, reproduced from [9], with permission from MDPI (Basel, Switzerland), 2019.

Mahata et al. reported an RRAM device with ALD-based HfO_2_/Al_2_O_3_ stack layers, which exhibited excellent performance with operation voltage lower than ~2 V and the ON/OFF ratio around ~10^3^. The TaN/HfO_2_/Al_2_O_3_/ITO RRAM device also presented neuromorphic synaptic behaviors with multi-level conductance properties by tuning the stop voltage in a DC sweep and the amplitude in pulse responses [20]. Shen et al. presented various RRAM devices with solution-processed AlO_x_ layers annealed at different temperatures [9]. As illustrated in Figure 7, their research results on Ni/solution-processed AlO_x_/Pt RRAM devices, indicating that the best performance can be found in the device with a solution-processed AlO_x_ layer annealed at 250 °C, which exhibited operation voltage around ~1 V, ON/OFF ratio higher than ~10^3^, endurance cycles over 100, and retention time longer than 10^4^ s. Samanta et al. reported the threshold switching performance of their cross-point selector with Al_2_O_3_ and SiO_x_ as bilayer dielectric [92]. Compared with the cross-point selector with only sputtering-deposited SiO_x_ layer, the ALD-deposited Al_2_O_3_ layer in the bilayer platform controlled the dissolution gap of Ag filament and improved the uniformity of the device. The selectivity was larger than 5 × 10^7^ and the rectifying ratio (RR) was over ~10^7^. Other researchers like Banerjee, Knorr and Sleiman et al. also demonstrated their investigation on RS behaviors of AlO_x_-based RRAM devices [93,94,95,96,97]. 

Kang et al. proposed a Ti/IL-NiO/Pt RRAM device with IL-treated NiO layer, which made the RRAM device operated under only ~0.5 V voltage bias with ON/OFF ratio higher than ~10^3^ [8]. The IL treatment on NiO thin film created Ni^0^-regions near the NiO/Pt interface and was helpful to the formation of oxygen vacancy filament, which improved device performance. The self-assembled memristive element based on NiO nanocrystal arrays was proposed by Kurnia et al. [98]. NiO nanocrystals were synthesized onto a SrRuO_3_ substrate with PLD (pulsed laser deposition) using the phase separation method. Their devices exhibited memristive switching behavior with nonlinear bipolar characteristics under the ~5 V operation voltage, which was investigated via scanning probe microscopy, based on first-order reversal curve current–voltage spectroscopy. In addition, it was indicated that low electrical dissipation at the edge of the nanocrystals represented that less energy was consumed as heat, which enhanced the utilization efficiency during the nucleation process of CFs and reduced the energy consumption. Besides, further exploration on RS performance of NiO dielectric was also carried out by Yoshida, Russo, Cagli, and Ielmini et al. [99,100,101,102,103].

Kim et al. reported an RRAM device with a structure of Au/TiO_x_/TiO_y_/Au, and the TiO_x_/TiO_y_ layers were fabricated by the sputtering method [78]. During the experimental process, their modified operations on the gas environment made TiO_x_ and TiO_y_ layers deposited under a gas mixture of Ar and O_2_ with flow ratios of 20:5 and 20:1, respectively. The Au/TiO_x_/TiO_y_/Au RRAM device operated under ~1.5 V with ~10^2^ ON/OFF ratio, which also exhibited artificial synaptic characteristics such as long-term potentiation (LTP), long-term depression (LTD), and spike-timing-dependent plasticity (STDP). Mullani et al. reported the enhanced RS behavior of their devices based on hydrothermal-fabricated carbon nanotube/TiO_2_ nanorods composite film through increasing oxygen vacancy reservoir [4]. The effect of concentration of TiO_2_-fMWCNT (functionalized multiwalled carbon nanotube) nanocomposites was confirmed, which improved the RS performance of the device with forming-free and low operational voltage when the concentration of fMWCNT was 0.03 wt %. Sakellaropoulos et al. demonstrated a comparison among different devices with three kinds of dielectric structures such as HfO_x_, TaO_y_/HfO_x_ and HfO_x_/TaO_y_/HfO_x_, which were corresponding to single-layer (SL), bilayer (BL) and triple-layer (TL) [104]. The forming-free sample TL exhibited enhanced RS behavior with ON/OFF ratio larger than ~10^2^, cycling variability smaller than 0.6 and number of endurance cycles over 10^6^ under only ~nW-level operation power in pulsing mode. Compared with sample SL, the RS performance of sample TL was confined due to the higher oxygen content and deeper oxygen vacancy levels of the TaO_y_ layer, which demonstrated analog switching characteristics and revealed great potential in artificial synaptic application. Besides, other researchers such as Nauenheim, Hermes, Salaoru and Otsuka et al. also provided a related investigation on TaO_x_- and TiO_x_-based RRAM devices in terms of electrical performance and physical characterization [105,106,107,108,109].

Wang et al. proposed an interface engineering method on ALD-based HfO_2_ thin film with O_3_, which improved the performance of Pt/HfO_2_/TiN RRAM device [79]. It is reported that the best stability could be observed in the HfO_2_ RRAM device with 20 pulses of O_3_ treatment. The TiON layer was observed at the interface between HfO_2_ and TiN layers. With the voltage bias onto electrode, more abundant detects survived at the TiON layer due to the longer oxidation process, which had a positive influence on the migration efficiency of oxygen vacancy and formation of CFs. However, it also resulted in the obvious augmentation of the conductivity of the HfO_2_ layer. In addition, their research on neuromorphic simulation based on potentiation, depression and STDP emulated the presynaptic and postsynaptic membranes of a biological synapse through applying the same pulses on both Pt and TiN electrodes, which revealed that oxide/metal interface engineering could have significant impact on RS characteristics of RRAM devices. Sharath et al. reported their RRAM devices based on RMBE (reactive molecular beam epitaxy)-deposited HfO_2-x_ layer with the operation voltage lower than 1 V and ON/OFF ratio higher than 10^2^ [110]. With the Hard X-ray photoelectron spectroscopy, the presence of sub-stoichiometric hafnium oxide and defect states near the Fermi level were confirmed. Bipolar RS performance was also observed on forming-free RRAM devices with oxygen-deficient HfO_2−x_ thin films. Besides, other related research on RS behaviors of HfO_x_ dielectric thin films were also reported by Clima, Lanza and Stefano et al. [111,112,113,114].

Ha et al. demonstrated an Ag/ZrO_2_/ITO RRAM device with sol-gel-processed ZrO_2_ thin film, which exhibited excellent performance with operation voltage around ~2 V and ON/OFF ratio higher than 10^5^ [22]. They investigated the effect of different annealing gas environments (air, vacuum and N_2_) on the performance of RRAM devices. Sol-gel-processed ZrO_2_ thin film annealed at vacuum showed enhanced performance with larger crystallinity and grain size, denser film, and a relatively small quantity of oxygen vacancies, which resulted in a decrease in the leakage current and an increase in the resistance ratio of HRS/ LRS and successfully improved non-volatile memory properties, such as endurance and retention characteristics. Abbas et al. proposed their investigation regarding Ti/ZrO_x_/Pt RRAM device with RTA (post-rapid thermal annealing) processing on ZrO_x_ dielectric layer [115]. Compared with samples based on an as-deposited ZrO_x_ layer without annealing process, RTA sample demonstrated improved RS performance such as lower operation voltage, higher RS ratio, longer retention time, and increasing number of endurance cycles. Particularly, with the annealing temperature of 700 °C during the RTA process, Ti/ZrO_x_/Pt RRAM device could operate with a voltage lower than 1 V and ON/OFF ratio higher than 10^3^. Apart from Ha and Abbas, Verbakel, Awais, Kärkkänen and Ismail et al. also exhibited their investigation on RRAM devices with ZrO_x_ dielectric layers and most of them fabricated ZrO_x_ thin films with ALD and sputtering methods [116,117,118,119,120].

Similar to binary metal oxides, binary nonmental oxides like SiO_2_ were also under investigation [49]. Yu et al. reported the multi-level RS performance of Ag/SiO_2_/Pt RRAM devices with operation voltage smaller than 1.5 V and switching ratio higher than 10^2^, which was affected by the formation of multiple Ag filaments [49]. Apart from binary oxides, complex oxides with higher dielectric constants, such as LaAlO_3_ [10,86], SrTiO_3_ [87,121], Pr_0.7_Ca_0.3_MnO_3_ [23,122] and BiFeO_3_ [11,88] are also explored to improve the switching performance of RRAM devices. Bailey et al. exhibited a stack-layered RRAM device with a structure of W/SrTiO_3_ (STO)/TiN, which systematically investigated the diffusion phenomenon of ionic defects in oxides associated with various configuration states of STO layer. Consistent decay was found during the test of device retention, and then the function relationship between decay rate and conditioning voltage was confirmed [121]. The metal/Pr_0.7_Ca_0.3_MnO_3_ (PCMO)/metal RRAM devices with a high-throughput electrode were studied by Tsubouchi et al. based on various metal electrodes including Mg, Ag, Al, Ti, Au, Ni, and Pt [122]. Typical RS behaviors were only observed on devices with Al electrode in the measurement process of I-V and pulsed-field resistance. In addition, the switching performance was always found near the Al/PCMO interface with the test results of the four-probe test. 

**Figure 8:**
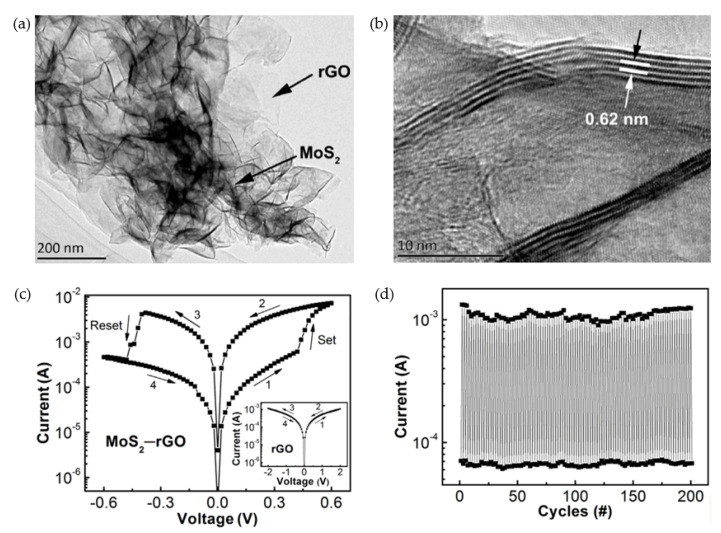
MoS_2_-rGO hybrid with (**a**) TEM and (**b**) HRTEM. (**c**) Bipolar I-V curves and (**d**) endurance properties of Ti/MoS_2_-rGO/ITO RRAM device, reproduced from [26], with permission from Elsevier, 2019.

Except binary and complex oxides, solid electrolyte and 2D materials are also popular in the research on RRAM-related inorganic materials. As early as in 1976, As_2_S_3_ solid electrolyte material has been studied as a functional layer in the RRAM device by Hirose et al. [123]. With the photodoping operation of Ag ions into the RS layer, the RRAM device with the Ag-As_2_S_3_ layer operated under low voltage, and CF based on the Ag element was observed by an optical microscope. After that, some solid electrolyte materials have been presented including Ag_2_Se [24], Ge_2_Sb_2_Te_5_ [25], GeTe [124], etc. At present, a series of 2D materials such as graphene [89,125,126], molybdenum disulphide (MoS_2_) [26,127,128] and perovskite materials (CH_3_NH_3_SnCl_3_ and CsPbBr_3_) [73,90] also inspired researchers’ interest due to their small size, ultra-thin thickness and excellent physical properties, which have resulted in superior performance of RRAM devices. Chen et al. proposed an electrode/oxide interface engineering technique by inserting single-layer graphene (SLG) into the TiN/HfO_2_/Pt RRAM device [89], which enabled the RESET current to be reduced by 22 times and the programming energy consumption reduced by 47 times. Raman mapping and single-point measurement on the SLG layer noted that signals of D-band and G-band changed during the electrical cycling, which indicated that oxygen ions might drift from metal oxide layer to SLG layer. Wu et al. exhibited a flexible RRAM device based on MoS_2_-rGO (reduced graphene oxide) hybrid layer synthesized by hydrothermal reaction between (NH_4_)_6_Mo_7_O_24_·4H_2_O and (NH_2_)_2_CS in GO (graphene oxide) dispersion with the distilled water as dispersant [26]. As illustrated in Figure 8, their device showed not only a low SET/RESET voltage (~0.4 V) but also multi-level states through compliance current (CC) control. Excellent electrical performance could also be observed with endurance cycles over 200, retention time longer than 10^4^ s and ON/OFF ratio higher than 10^3^. Many parameters about RS performance of RRAM devices were compared in this review, which can be also observed in Table 1.

### 3.2. Thin Film Materials of Electrode

Although materials application of RS medium has played a decisive role in performance of RRAM device, the effect of electrode thin film materials on devices cannot be neglected, which act as primary transport paths for carriers. For now, a great number of thin film materials have been investigated for the selection of electrode, such as elementary substance metals, carbon-based materials, conductive oxides, complex nitrides, and silicon-based materials. Table 2 demonstrated devices with various electrodes. During the research and selection of different materials for electrode of RRAM devices, some vital factors should be under special consideration, such as the activity of materials, work function of materials and the interface between electrode and RS layer [48]. 

**Figure 9:**
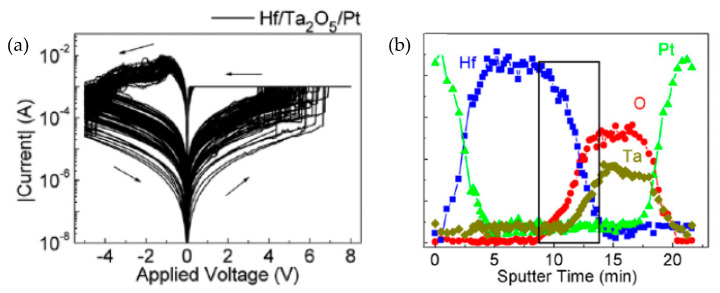
(**a**) Bipolar RS performance and (**b**) AES depth profiles of Hf/Ta_2_O_5_/Pt RRAM devices, reproduced from [129], with permission from AIP Publishing, 2013.

In general, the elementary substance metals are the most common and widely applied in RRAM devices, including Hf [129], Al [27], Ti [8], Zr [129], Cr [27], Ni [9], Cu [10], Ag [11], Pt [12], and Au [78]. Most of the time, RRAM devices always exhibit unipolar RS performance with noble metal electrode, such as Pt and Ru both as TE and BE [48,88]. However, the bipolar RS behavior will be always observed when one of the noble metal electrodes is replaced by an active metal electrode like Ni and TiN [8,85]. Chen et al. reported a Ta_2_O_5_-based RRAM device with metal Hf as TE and they covered a 50-nm-thick Pt layer onto the Hf layer to prevent the oxidation [129]. The Pt/Hf/Ta_2_O_5_/Pt device operated under ~4 V with ON/OFF ratio higher than 10^3^, which resulted from the fact that a higher Ta atomic concentration was observed at the Hf/Ta_2_O_5_ interface than that at the Ta_2_O_5_/Pt interface. It is further indicated that considerable diffusion of oxygen ions occurred in the Hf electrode and the obvious degeneration of O/Ta ratio occurred at the Hf/Ta_2_O_5_ interface, which can be illustrated in Figure 9. A similar structure was also reported to replace Hf with Zr as TE in Pt/Zr/Ta_2_O_5_/Pt device, which showed lower operation voltage than that of the Pt/Hf/Ta_2_O_5_/Pt device. Shen et al. proposed a GO-based RRAM device with Al and p-type Si as TE and BE, respectively [27]. Their RRAM device with solution-processed GO dielectric layer exhibited excellent performance with SET/RESET voltage lower than 2 V and ON/OFF ratio higher than 10^2^, which was affected by the diffusion of oxygen ions between metal Al and GO thin film. The Cr/Gd_2_O_3_/TiN RRAM device was proposed by Jana et al. [130]. The bipolar RS characteristics demonstrated that the RRAM device could operate with operation voltage lower than 1.5V and ON/OFF ratio higher than 10^4^. This 9-nm-thickness device exhibited excellent repeatable RS cycles with number of endurance pulses over 10^5^ and retention time longer than 3 × 10^4^ s. In addition, some RRAM devices with noble metal as both TE and BE also demonstrated bipolar switching performance, such as Pt/sputtering-deposited Cr_2_O_3_/Pt reported by Chen et al. [132], Pt/sputtering-deposited TaO_x_/Pt reported by Huang et al. [133], Pt/Ni/ECD (electrochemical deposition)-fabricated CuO_x_/Ni/Pt, and Pt/sputtering-deposited SiO_2_/Pt reported by Park et al. [134].

Apart from elementary substance metals mentioned, carbon-based materials such as graphene and carbon nanotubes [48,62,131,135], conductive oxides such as ITO (indium tin oxide) [131], and complex nitrides such as TiN and TaN [20,85] are also charming as selection of RRAM electrodes. ITO electrode has been widely utilized in electronic devices as well as RRAM cells due to its excellent electrical conductivity, high light transmittance and high hardness [48]. In addition, as one of the n-type semiconductors with highly degeneration characteristic, the ITO electrode has good electrical conductivity due to internal carriers including oxygen vacancy and activated Sn^4+^ ions [48]. Zhao et al. proposed an RRAM device with multilayer graphene (MLG) and ITO as TE and BE, respectively [131]. Their transparent MLG/Dy_2_O_3_/ITO device exhibited 80% transmittance at 550 nm wavelength due to the good light transmittance of the ITO electrode. The excellent electrical conductivity of graphene and ITO made the devices operate under low CC (<100 μA) with low voltage (<1 V) and low power consumption (<100 μW). The device also demonstrated a resistance ratio between HRS and LRS higher than 10^4^ during 200 successful switching cycles and retention longer than 10^4^ s. 

The significance of selecting electrode materials is to optimize the performance of RRAM devices. Electrode materials like inert metals (Pt and Pd) are always investigated as carrier transportation paths, which have a slight and limited influence on RS performance. Then, electrodes of an RRAM device should have a positive impact on the formation process of CFs, which resulted from the migration of anion, which is always observed in oxygen-vacancy-based RRAM devices [8,9,32]. Finally, the selection of electrode materials for RS medium can directly affect the concentration of migrated anion and accumulated vacancy/cation; proper choice of electrode materials will have a positive influence in the formation process of CFs and also achieve stability enhancement of RRAM device. 

## 4. Bionic Synaptic Application

**Figure 10:**
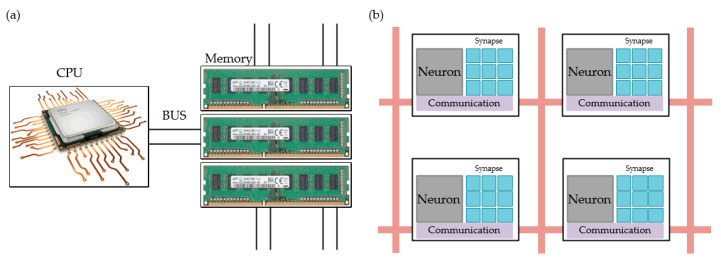
(**a**) Computing systems based on traditional Von Neumann architecture, the memory address of program instruction and the memory address of data point to different physical locations in the same memory device. (**b**) Computing systems based on neuromorphic architecture with integration of a single synaptic device into each unit.

With the rapid development of information technology, the neuromorphic computing applications in terms of hardware and software have stepped into the new era. For now, the neuron complexity simulation has been achieved with conventional computing technology based on animal brains of cats and rats [136,137]. However, as illustrated in Figure 10, it has been considered to be inefficient to process huge amounts of data with traditional computing architecture like Von Neumann architecture. The Von Neumann architecture was a binary-based computer concept structure proposed by John von Neumann in 1946, which indicated that a program should be considered as a part of data firstly [136,137]. Based on this architecture, the program itself and the data it processes should be stored with the same memory technology. The memory address of program instruction and the memory address of data should point to different physical locations in the same memory device [138,139]. Besides, the width of program instruction should be the same as that of processed data. As illustrated in Figure 10a, programs in matrix multiplication always process a lot of data at a slow speed, and the speed is slowed down with the increase of hidden layers. Energy power consumption also increases at the same time. Compared with CPU and GPU-based computer systems, although emerging dedicated FPGA and ASIC hardware chips have demonstrated faster operation speed and lower energy consumption, the matrix multiplication for large amounts of data is still limited by operation speed and energy consumption. 

Compared with a conventional computer system based on Von Neumann architecture, artificial neural networks (ANNs) have received extensive attention due to their lower power consumption and higher operation efficiency [138,139]. However, conventional synaptic devices cannot meet the requirements of ANNs because of some technological limitations like large device area, high power consumption and slow response speed. As illustrated in Figure 10b, emerging synaptic device can be integrated in to a single unit and improves the processing efficiency and processing accuracy [15,17,34,35,36,37,38]. The superior RS characteristics demonstrated in RRAM devices reveal its great potential in the neuromorphic application based on ANNs. An RRAM device can store multi-bit information in a non-volatile manner on a 4F^2^ device area and can also switch with the energy of ~pJ-level, which enables significant improvements in high-density integration and low power operation compared to conventional CMOS-based synapse devices [135,136,137,138].

**Figure 11:**
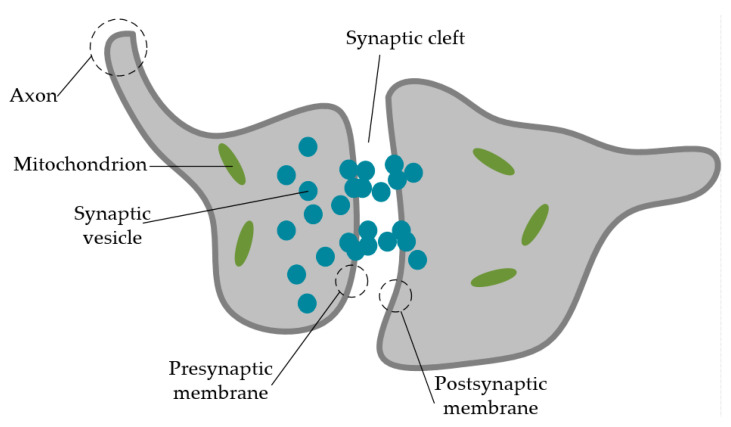
Structure of synapse in neural network.

In the analog circuit of ANNs, RRAM devices act as substitution to synapses in order to provide connection function between neurons and information storage cells [140,141]. The ultra-small size of RRAM device is to increase the synapse density of ANNs, which is expected to reach the synapse density in human brain (~10^10^ synapses/cm^2^) [28,142]. As the key role during the information delivery process, a synapse is responsible for transmitting impulses from one neuron to another, which is considered to provide dynamical interconnections between two connecting neurons. Figure 11 demonstrated the basic structure of biological synapse, which mainly includes presynaptic membrane, synaptic cleft, postsynaptic membrane, and neurotransmitter [143]. Presynaptic membrane and synaptic vesicle are included by the presynaptic element, which is the dendrite/axon terminal of pre-neuron. Chemical substances in synaptic vesicle are called neurotransmitters. With the external spiking or stimulation onto the presynaptic element, synaptic vesicle filled with neurotransmitter will be released from presynaptic membrane to postsynaptic membrane due to the flux of Ca^2+^, which has a temporary influence on synaptic transmission. The transmitting process of the neurotransmitter through a synapse represents the information delivery among neurons. The gap between presynaptic and postsynaptic membranes is the synaptic cleft, which is around 15~30-nm thick [144,145]. In general, for application in neuromorphic system and ANNs, capacitor-like synaptic devices are always used to simulate neuron bodies in the human brain; a neuron in the human brain can be compared to an electronic device with combined functions including an integrator and a device for threshold spiking. An axon acts like a connecting bridge in order to provide a transmission connection of information, which can be considered as a long wire. A dendrite works to transmit the signal input from multiple neurons to a single neuron, which can be compared to a short wire. A synapse acts as a connector to provide connection function between two next neurons [2], which have been attached to the most interests for now.

Massive neurons form neural circuits through synapses in neural networks of the human brain [1,2,3,4,5,6]. These neural circuits are the foundation of advanced neural activities such as learning and memorizing, which are attributed to the plasticity of neural networks [2,3,4]. Basically, the plasticity of neural network is corresponding to the synaptic plasticity, which is dependent on synaptic weight [146,147]. Synaptic weight is used to demonstrate the strength or amplitude of connection between the two next neurons, which is mainly related to the amount of released or absorbed neurotransmitter. According to the theory proposed by Hebbian in 1949 [148], the connection between the two next neurons can be strengthened when they receive stimulation signals, which will result in the potentiation and depression during the neuron activities. For instance, long-term potentiation is corresponding to the synaptic potentiation behavior while long-term depression indicates depression performance of synapse. The synaptic plasticity mainly induced by the change of synaptic weight is divided into long-term plasticity (LTP) and short-term plasticity (STP) [149,150]. STP includes short-term potentiation, pair-pulse facilitation (PPF) and depression while long-term potentiation and depression are considered as representations of LTP [151]. For a biological neural system, on the one hand, STP is responsible for critical computation, on the other hand, LTP is thought of as the foundation of ability in terms of machine learning and memorizing [151]. As the molecular mechanism for learning and memorizing in the human brain, the timescale of STP can sustain from tens of milliseconds to few minutes, while the retention time of LTP can be over a few hours, even several days. The transition from STP to LTP can be achieved through repeated stimulation during the learning process. Apart from LTP and STP, the plasticity depends on spike time (spike-time-dependent plasticity, STDP) is also under investigation, which is one of the advanced learning characteristics in the neuron system of the human brain [152,153]. As indicated by Hebb’s rule, when the presynaptic membrane is stimulated earlier than the postsynaptic membrane and the postsynaptic current can be enhanced, which is called long-term potentiation. Reversely, when the spike occurs on the postsynaptic membrane earlier than that on the presynaptic membrane, the postsynaptic current will be depressed, namely long-term depression. In addition, it is noted that with the change of relative time between two stimulations (commonly pulses), the postsynaptic current will also be influenced [154]. Due to the existence of RS characteristics, the resistance states of RRAM devices can be manipulated by applied voltages and the microscopic conductive paths through the RS medium will be acquired. Therefore, resistance values of RRAM devices are the response to periodic input signals (voltage or current), which can be considered as the synaptic weights. The modification operations of resistance represent the changes of synaptic weights. When the external power supply fails to work, resistance states can also be retained [2]. 

### 4.1. Short-Term Plasticity for RRAM Devices

**Figure 12:**
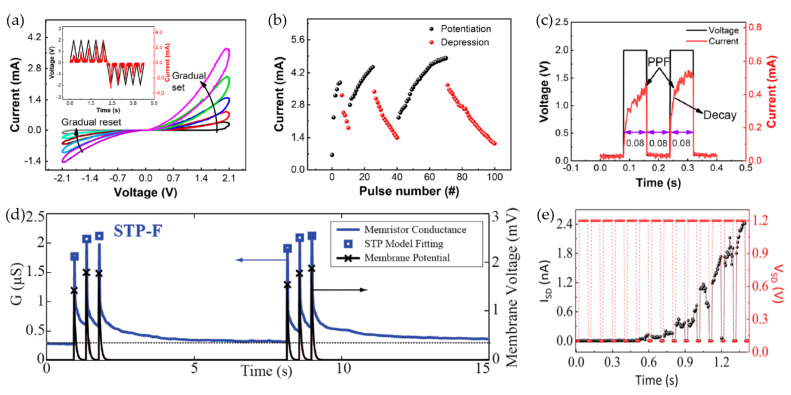
(**a**) Potentiation and depression response of Ag/SiO_x_:Ag/TiO_x_/p^++^-Si device with repeated voltage sweeps. (**b**) Conductance modulation and (**c**) PPF of Ag/SiO_x_:Ag/TiO_x_/p^++^-Si device by repeating consecutive pulses. (**d**) Repeated STP response with the model fitting of TiO_x_-based RRAM device, reproduced from [158], with permission from Springer Nature, 2020. (**e**) Synaptic facilitation response to consecutive pulses of the device with h-BN, reproduced from [159], with permission from Elsevier, 2020.

In general, two types of RS behaviors observed in RRAM devices, abrupt and gradual RS behaviors, were considered corresponding to digital and analog switching, respectively. The abrupt performance for resistance change was believed to be consistent with a digital signal while the gradual one with continuous conductance changes showed similar characteristics like a biological synapse [155]. Ilyas et al. demonstrated their research on a physical-vapor-deposited Ag/SiO_x_:Ag/TiO_x_/p^++^-Si RRAM device with bilayer dielectric in terms of STP such as potentiation, depression and PPF [155]. The conductance of this bilayer device was modified by positive and negative pulses. As illustrated in Figure 12a,b, with the repeated voltage sweep, the potentiation and depression behaviors were observed and indicated the conductance states change during the processes of potentiation and depression, which proved the feasibility of conductance modification and simulated the change of synaptic weight. Figure 12c demonstrated the results of PPF for this bilayer device. PPF is always investigated to provide adjustment for conductance in order to perform short-term neural behaviors such as synaptic filtering and adaptation [156,157]. During two continuous pulse stimulations of their Ag/SiO_x_:Ag/TiO_x_/p^++^-Si samples, the next post-synaptic response became higher than that of the previous one, which indicated that the interval time of spike was less than the recovery time. Berdan et al. also reported the STP of TiO_2_-based RRAM devices through the transient conductance response [158]. As illustrated in Figure 12d, the conductance of the device increased due to the applied pulse and then decayed back to its initial state slowly. A similar performance could be observed on subsequent pulses who were dependent on the previous resistance states. Apart from RRAM devices with traditional metal oxides, short-term synaptic performance of devices fabricated with 2D materials are also under investigation [159,160,161]. Sun et al. reported their research on 2D-material-based devices with h-BN as a functional layer [159]. As demonstrated in Figure 12e, an obvious increase could be observed for ON-state current, which indicated the rise of synaptic strength due to the repeated pulse stimulation. The short-term facilitation with applied voltage-pulse excitation was observed on Ag/h-BN/graphene device as well. Sokolov et al. also provided their investigation results about PPF response of RRAM devices based on TaO_x_/IGZO (InGaZnO) bilayers [162]. Firstly, the single pulse with 0.75 V amplitude and 2 ms width was applied onto TE of RRAM device and the device exhibited a corresponding current response of ~220 mA. After that, with the RESET operation, the device transferred to HRS with initial OFF-state current. Then, two consecutive stimuli equal in amplitude and width to the first single pulse were applied onto TE, and the current response was ~350 mA, which indicated that the TaO_x_/IGZO-based RRAM device could enhance the trans-conductance with the effect of high-frequency stimuli and further improve the PPF phenomenon. 

### 4.2. Long-Term Plasticity for RRAM Devices

**Figure 13:**
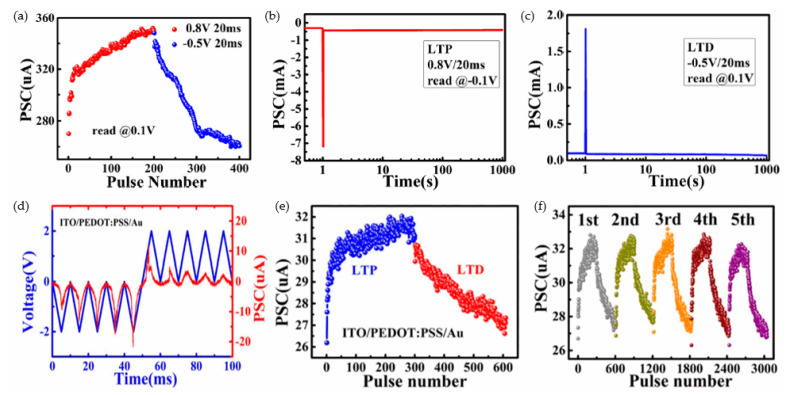
(**a**) Gradual modulation for conductance with long-term potentiation/depression response of Ag/HZO/ITO/PET RRAM device. Retention performance of Ag/HZO/ITO/PET RRAM device in (**b**) long-term potentiation process by consecutive positive pulses and (**c**) long-term depression process by consecutive negative pulses, reproduced from [79], with permission from Springer Nature, 2019. (**d**) Synaptic weights modulation of Au/PEDOT:PSS/ITO RRAM device by 10 consecutive pulses. (**e**) Long-term potentiation/depression under 600 consecutive pulses in one operation and (**f**) five operations of long-term potentiation/depression for Au/PEDOT:PSS/ITO RRAM device, reproduced from [163], with permission from MDPI (Basel, Switzerland), 2018.

Compared with the investigation of STP, current research on LTP mainly focuses on long-term potentiation/depression and transition from STP to LTP [79,163,164]. Wang et al. proposed a flexible bipolar RRAM device with ALD-deposited Hf_0.5_Zr_0.5_O_2_ (HZO) dielectric layer, as illustrated in Figure 13a–c, which worked as artificial synapses in the neuromorphic network in order to overcome the bottleneck based on traditional Von Neumann structure [79]. During the 400 continuous programming pulses, devices with gradual RS behaviors in DC sweep exhibited the feasibility of conductance modification under a sequence of consecutive programmable pulses, which indicated that the synaptic devices that emulated long-term potentiation/depression had great potential of artificial application in a neuromorphic computing system. The potentiated and depressed performance were stimulated by applying 200 0.8 V/20 ms continuous pulses and 200 0.5 V/20 ms continuous pulses in Figure 13a, respectively. This Ag/HZO/ITO/PET RRAM device showed the decayed post-synaptic current (PSC), and the PSC state turned into intermediate over time due to the forgetting effect. Figure 13b,c showed excitatory and inhibitory states of PSC with a single pre-synaptic spike, and the retention time over 1000s demonstrated the reliability of STP in Ag/HZO/ITO/PET RRAM devices. Wang et al. demonstrated their research report on a flexible RRAM device fabricated with a common polymer, poly (3,4-ethylene dioxythiophene): poly (styrene sulfonate) (PEDOT:PSS), as functional layer [163], and the PEDOT:PSS solution was spin-coated onto the ITO substrate. They chose consecutive positive and negative voltage bias carefully to apply onto TE Au of their device in order to avoid abrupt RS performance. The decreased conductance was observed when the continuous positive voltage bias swept from 0 V → 3 V → 0 V and then the gradual increased conductance was demonstrated with the consecutive negative voltage bias from 0 V → −2 V → 0 V, which indicated the potential of successful modulation of synaptic weights. The consecutive pulses with 2 V amplitude and 10 ms width were applied to TE for observing the current response, as demonstrated in Figure 13d; the potentiation and depression responses were observed with five consecutive positive pulses bias and five consecutive negative pulses bias, respectively. After that, the Au/PEDOT:PSS/ITO RRAM device was applied consecutive pulses (300 1V/10 ms positive pulses and 300 −1.5 V/10 ms negative pulses) and the long-term potentiation and depression were emulated successfully in Figure 13e. Finally, Wang et al. repeated the pulse trains five times, as illustrated in Figure 13f, which indicated the excellent endurance property of their device. 

**Figure 14:**
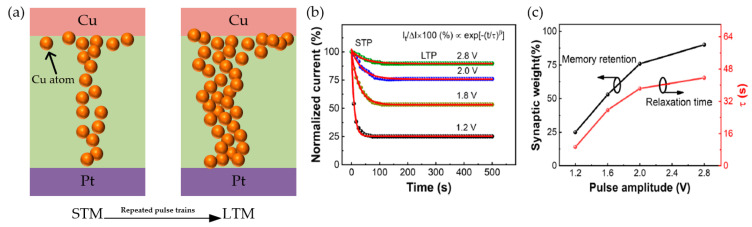
(**a**) Illustration of Cu atom dynamics of Cu/a-Si/Pt device during the transition from STM to LTM. (**b**) Relationship between normalized current response and retention time when the transferring process from STP to LTP occurred in Ag/SiO_x_:Ag/TiO_x_/p^++^-Si device. (**c**) Synaptic weight response to changes of pulse amplitude and relaxation time τ, reproduced from [155], with permission from Springer Nature, 2020.

Except for the research on long-term potentiation and depression behaviors of RRAM devices, the transition from STP to LTP also attracted significant interests [151,155,165], which is corresponding to transition from short-term memory (STM) to long-term memory (LTM). Biologically, compared with LTP, the sustainment time of STP is shorter generally, which is related to forgetting behaviors in the human brain. However, the STP can transfer into LTP with the repeated stimuli or a series rehearsal operation [155]. Zhang et al. reported the mechanism of conversion from STM to LTM in Cu/a-Si/Pt RRAM device [151]. As illustrated in Figure 14a, there were little Cu^2+^ ions drifting into the RS layer with a few stimulations, and then CF based on Cu atoms spontaneously decayed back to the initial state. However, with more repeated stimulations onto TE Cu, more Cu^2+^ ions migrated to the RS layer, which showed similar performance like training operation in the neural network. Ilyas researched the transition from STP to LTP of Ag/SiO_x_:Ag/TiO_x_/p++-Si device through modulating pulse strength [155]. Figure 14b,c showed the normalized current response by applying pulses with various amplitudes (1.2 V, 1.8 V, 2.0 V and 2.8 V) and each training cycle included 50 pulses. With the experimental results and the fitting results based on the equation in Figure 14b, the increased tendency of relaxation time was confirmed due to the effect of pulse strength (the red line in Figure 14c). An obvious elevation of synaptic weight was also observed when the relaxation time was around 45 s and the pulse amplitude was +2.8 V, which indicated that STP has transferred to LTP.

### 4.3. Spike-Time-Dependent Plasticity for RRAM Devices

Currently, many research achievements have proved that long- and short-term plasticity functions of biological neural synapse could by mimicked by RRAM devices with modulation of applied pulses (including amplitude and number modulations). On the other hand, as one of the determining factors in synapse plasticity, STDP also has received extensive attention. STPD can be defined as a function relationship between change of synaptic weight (ΔW) and time interval (Δt) resulting from activity variation of the pre- and post-neurons [155,163]. When Δt > 0, the activity of pre-synaptic neuron precedes that of post-synaptic neuron, which means that the connection strength between these two neurons will be reinforced and result in the long-term potentiation of the synapse. Conversely, the long-term depression occurs when the spike of post-synaptic neuron heads that of pre-synaptic neuron and the connection strength is weakened (Δt < 0). Obviously, for an RRAM device, top and bottom electrodes are compared to pre- and post-synaptic neurons and pulses applied onto electrode can mimic spikes of biological synapses. The polarity of ΔW is determined by the order of spikes of pre- and post-synaptic neurons. In general, the time interval and change of synaptic weight are defined as follows:

(6) $$\Delta t=t_{post}-t_{pre}$$

(7) $$\Delta W=\frac{G_{t}-G_{i}}{G_{i}}\times 100\%$$

in which $t_{pre}$ and $t_{post}$ are time nodes of spikes applied onto pre- and post-synaptic neurons, respectively. $G_{i}$ is the conductance of the device at the initial state when t is 0, and $G_{t}$ is the conductance when the time reaches node t. 

**Figure 15:**
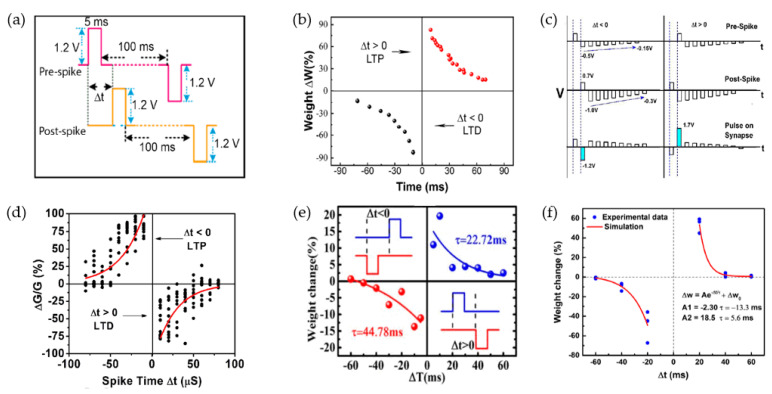
(**a**) Implementing programming pulses and (**b**) STDP behavior of Ag/SiO_x_:Ag/TiO_x_/p^++^-Si RRAM device, reproduced from [155], with permission from Springer Nature, 2020. (**c**) Applied pre/post-spikes with sequences and (**d**) STDP characteristics of TaN/HfO_2_/Al_2_O_3_/ITO RRAM device, reproduced from [20], with permission from Elsevier, 2020. (**e**) STDP results with pulse interval modulation of pre- and post-synaptic spiking for PEDOT:PSS-based RRAM device, reproduced from [163], with permission from MDPI (Basel, Switzerland), 2018. (**f**) Experimental and fitting results of STDP behaviors for Ag/SrTiO_3_/RGO/FTO RRAM device, reproduced from [166], with permission from Elsevier, 2018.

Ilyas et al. emulated the STDP rule of Ag/SiO_x_:Ag/TiO_x_/p^++^-Si samples, which can be observed in Figure 15a,b [155]. Through the implementation of a pair of pulses ±1.2 V/5 ms, ΔW decreased with the increase of Δt, which indicated that a more obvious conductance change could be observed when the time interval decreased. When Δt > 0, the pre-spike occurred before the post-spike, and the increased ΔW proved the enhancement of device conductance along with the decreasing Δt. Inversely (Δt > 0), the depression of device conductance was observed in ΔW when Δt increased. Mahata et al. revealed the STDP characteristic of RRAM devices with bilayer metal-oxide dielectric (TaN/HfO_2_/Al_2_O_3_/ITO) [20]. As illustrated in Figure 15c,d, a series of pulses with different amplitudes were applied on to TaN and ITO electrodes. The largest values of ΔW were 97% and −84% when the |Δt| was 10 μs at both states (Δt > 0 and Δt < 0), which confirmed that their bilayer RRAM devices had STDP behaviors at various spiking timings. Wang et al. reported the STDP behaviors of the RRAM device fabricated with organic 2D materials [163]. After the test of long-term potentiation and depression with 600 consecutive programming pulses, they used a pair of pulse (±1.5 V/10 ms) to provide spikes on TE and BE of device, and the related result can be observed in Figure 15e. When the pre-synaptic neuron was spiked earlier than the post-synaptic neuron, the potentiated response of connection strength between two neighboring neurons could be demonstrated. On the contrary, the decreased synaptic weight indicated the weak connection. Wan also reported the STDP of 2D-material-based RRAM with the structure of Ag/SrTiO_3_/RGO/FTO [166]; a similar experimental response with applied pulses (±1 V/10 ms) was observed in Figure 15f. The relationship between time interval and change of synaptic weight was similar to results proposed by others, which was more similar to their fitting results. These results proved that a more considerable change of synaptic weight could be realized with the smaller time interval of activity between two adjacent synaptic neurons. 

## 5. Conclusions

In this work, we have provided an overview of RRAM devices with advances including various thin film materials applied in RS layer and electrode, classification of RS mechanisms and investigation on artificial synapse. Many research reports indicate that RRAM devices fabricated with inorganic materials, such as oxides, solid electrolyte and two-dimensional (2D) materials, have demonstrated relatively mature performance. There is great potential for the application of organic materials (biological and polymer materials) in RRAM devices accordingly. The performance of the devices depends largely on the RS mechanisms, which also has a strong connection with choice and processing techniques of the thin film materials. Based on the fundamental performance of RRAM devices, some outstanding enterprise or research institutions such as Samsung Electronics, Intel Corporation and Institute of Microelectronics of the Chinese Academy of Sciences (IMECAS) have put effort into promoting the development of large-scale manufacture and mature product commercialization of RRAM devices for many years. As early in 2004, Samsung Electronics reported the highly scalable TMO (binary transition-metal-oxide) RRAM devices with CMOS technology in IEDM (International Electron Devices Meeting) and NiO was used as a functional layer [167]. In 2007, the 2-MB CBRAM test chip was reported by Infineon and Samsung exhibited the 3D RRAM array with 1D1R structure [2]. From 2010 to 2013, Unity Semiconductor Corporation firstly reported their 64-MB RRAM test chip and SanDisk/Toshiba reported their 32-Gbit bilayer RRAM test chip. They realized the practical experiment in electrical circuits with devices fabricated by TaO_x_ and HfO_x_ functional layers [168]. One year later, Micron/Sony presented their 27-nm 16-Gbit CBRRAM test chip and TaO_x_/HfO_x_ functional layers with stack structure were investigated [169]. In 2016, the four-layer 3D vertical RRAM array with self-selecting characteristic was reported by IMECAS, and RS performance of HfO_x_ resistive layers with the multi-level structure was verified [170]. In 2019, Intel Corporation announced they had prepared to manufacture emerging RRAM devices with 22 nm process technology, which also indicated that binary metal oxides or perovskite materials might be considered as candidates for the selection of functional layers [171]. All these developments are proving that the potential of large-scale commercialization for RRAM technology with different materials (especially TMO) is enormous and promising. Apart from the traditional large-scale commercialization process, the final objective of investigating different RRAM device performances is to provide potential assistance to artificial intelligence and neuromorphic computing systems. RRAM devices can mimic functions of biological synapse with electrical performance, which has a positive influence in hardware application of the artificial intelligence field. In addition, its human-brain-like behaviors such as STM and LTM make the development of neuromorphic computing system possible in the coming future.

## Figures and Tables

**Table 1:** Performance comparison among RRAM devices with various binary and complex oxides (metal and nonmetal materials).

Structure	Switching Mode	Thickness (nm)	V_Forming_ (V)	V_SET_ (V)	V_RESET_ (V)	ON/OFF Ratio	Endurance (cycle)	Retention (s)	Ref.
Ni/AlO_x_/Pt	bipolar	~40	Free	~1.0	~−1.0	~10^3^	>150	>10^4^	[9]
TaN/HfO_2_/ Al_2_O_3_/ITO	bipolar	~6	~4.5	~1.5	~−1.0	~10^2^	>100	>2 × 10^3^	[20]
Ti/IL-NiO/Pt	bipolar	~50	Free	~0.5	~−1.5	~10^3^	>1300	>10^4^	[8]
FeNi/Al_2_O_3_/NiO/Pt	bipolar	~180	~4.07	~6.0	~−5.0	~10^3^	>100	>10^4^	[12]
Au/TiO_x_/ TiO_y_/Au	bipolar	~50	~5.62	~1.0	~−2.0	~10^2^	N. A.	N. A.	[78]
Ni/SiGeO_x_/ TiO_y_/TiN	bipolar	~25	Free	~3.0	~−2.5	~10^3^	>10^4^	>10^5^	[21]
Ti/HfO_2_/TiN	bipolar	~15	~6.5	~1.0	~−0.8	~10	N. A.	N. A.	[60]
Pt/Hf/HfO_2_/TiN	bipolar	~20	Free	~0.8	~−1.5	~10^2^	N. A.	>10^6^	[80]
Pt/Ta/HfO_2_/TiN	bipolar	~20	Free	~0.8	~−1.8	~10^2^	N. A.	>10^4^	[80]
Pt/Al:HfO_2_/TiN	bipolar	~9	~2.3	~2.0	~−2.0	~10^4^	>100	>10^4^	[32]
TiN/ZnO/ TiN	bipolar	~9	~4.2	~1.0	~−1.0	~10	240	N. A.	[82]
TiN/Al_2_O_3_/ ZnO/Al_2_O_3_/TiN	bipolar	~15	~5.0	~1.0	~−1.0	~10^2^	>10^4^	>10^4^	[82]
ITO/ZrO_2_/Ag	bipolar	~50	N. A.	~5.0	~−15.0	~10^5^	>100	>10^4^	[22]
Pt/N:ZrO_2_/ TiN	bipolar	~25	~3.6	~0.5	~−1.0	~10^2^	N. A.	N. A.	[85]
Ag/SiO_2_/Pt	bipolar	~80	N. A.	~0.5	~−2.0	~10^6^	>40	>2 × 10^3^	[49]
ITO/LaAlO_3_/ITO	bipolar	~30	~3.2	~3.0	~−3.0	~10^2^	>100	N. A.	[86]
Cu/Cu:LaAlO_3_/Pt	bipolar	~10	~7.0	~2.0	~−2.0	~10^3^	>110	>10^4^	[10]
GNR/SrTiO_3_/GNR	bipolar	~50	N. A.	~2.0	~−3.0	~10	>200	>10^4^	[87]
Pt/GO/PCMO/Pt	bipolar	~25	Free	~1.0	~−1.0	~10^2^	>150	>10^4^	[23]
Pt/BiFeO_3_/Pt	unipolar	~200	N. A.	~5.0	~−15.0	N. A.	N. A.	N. A.	[88]
Ag/ZnO/BiFeO_3_/ZnO/Ag	bipolar	~270	Free	~2.0	~−2.0	~10	N. A.	N. A.	[11]
Ag/Ag_2_Se/ MnO/Au	bipolar	~40	Free	~0.8	~−0.6	~10^2^	>800	>10^4^	[24]
TiN/SLG/HfO_2_/Pt	bipolar	~35	~5.0	~2.0	~−3.0	~10^2^	>120	>10^6^	[89]
Ti/MoS_2_-rGO/ITO	bipolar	~60	Free	~0.5	~−0.4	~10	>200	>10^4^	[26]
Au/CsPbBr_3_/ITO	bipolar	N, A.	Free	~1.0	~−1.0	~10^4^	N. A.	>1200	[90]

**Table 2:** RRAM devices with various materials as electrodes.

Electrode Materials	Thickness (nm)	Electrode Mode	Switching Mode	V_SET_ (V)	V_RESET_ (V)	ON/OFF Ratio	Ref.
Hf	~40	TE	Bipolar	~4	~-4	~10^3^	[129]
Al	~40	TE	Bipolar	~2	~-2	>10^2^	[27]
Ti	~100	TE	Bipolar	~0.5	~-1.5	~10^3^	[8]
Zr	~40	TE	Bipolar	~2	~-4	~10^3^	[129]
Cr	~70	TE	Bipolar	~1.5	~-1.5	~10^4^	[130]
Ni	~40	TE	Bipolar	~1.0	~-1.0	~10^3^	[9]
Cu	~150	TE	Bipolar	~2.0	~-2.0	~10^3^	[10]
Ag	~140	TE/BE	Bipolar	~2.0	~-2.0	~10	[11]
Pt	~200	BE	Bipolar	~6.0	~-5.0	~10^3^	[12]
Au	~50	TE/BE	Bipolar	~1.0	~-2.0	~10^2^	[78]
Graphene	N. A	TE	Unipolar	~1.0	~-1.0	~10^4^	[131]
ITO	~50	BE	Bipolar	~1.5	~-1.0	~10^2^	[20]
TiN	~20	TE	Bipolar	~0.5	~-1.0	~10^2^	[85]
TaN	~60	TE	Bipolar	~1.5	~-1.0	~10^2^	[20]
p-type Si	~100	BE	Bipolar	~2	~-2	>10^2^	[27]
